# *PDCD1* SNPs and alloimmune transplant outcomes: mechanistic basis and human evidence across solid organ transplantation and allogeneic hematopoietic stem cell transplantation

**DOI:** 10.1016/j.bbrep.2026.102807

**Published:** 2026-09-23

**Authors:** Hosein Rabet, Sayed Mostafa Hosseini

**Affiliations:** a Student Research Committee, Baqiyatallah University of Medical Sciences, Tehran, Iran; b Chemical Injuries Research Center, Systems Biology and Poisoning Institute, Baqiyatallah University of Medical Sciences, Tehran, Iran

**Keywords:** PDCD1, PD-1/PD-L1 axis, Single-nucleotide polymorphism, Transplant rejection, Solid organ transplantation, Allogeneic hematopoietic stem cell transplantation, Graft-versus-host disease, Immune checkpoint

## Abstract

**Background:**

The programmed cell death 1 gene (*PDCD1*) encodes programmed death-1 protein (PD-1), an inhibitory immune-checkpoint receptor that regulates peripheral tolerance and alloimmune activation.

**Scope:**

This narrative review synthesizes mechanistic and human evidence linking *PDCD1* single-nucleotide polymorphisms (SNPs) to transplant-related alloimmune outcomes. In solid organ transplantation (SOT), outcomes are framed mainly as rejection-related endpoints, whereas in allogeneic hematopoietic stem cell transplantation (allo-HSCT), bone marrow transplantation (BMT), and cord blood transplantation (CBT), graft-versus-host disease, relapse, infection, survival, mortality, and engraftment are treated as clinically distinct endpoints rather than SOT-like rejection.

**Evidence synthesis:**

*PDCD1* variants encompass regulatory, intronic, coding, and 3′UTR loci with potentially different biological consequences. For some variants, experimental studies support effects on transcriptional regulation or transcription-factor binding, whereas for others the biological significance remains uncertain and may depend on linkage disequilibrium with neighboring regulatory alleles. Human transplant studies remain limited and heterogeneous: SOT studies are largely negative or inconclusive, whereas allo-HSCT/CBT studies report associations with GVHD, relapse, infection, survival, mortality, or engraftment, most of which await independent replication. Accordingly, the reported genotype–outcome relationships should be regarded as associations rather than evidence of causality, and the current evidence is insufficient to support validated clinical prediction based on individual *PDCD1* variants.

**Conclusion:**

*PDCD1* variation is biologically plausible as a context-dependent modifier of alloimmune outcomes, but no individual variant is ready for clinical risk prediction. Future studies should prioritize transplant-specific endpoints, paired donor-recipient analyses, and independent validation.

## Highlights

• PDCD1 variants may tune alloimmune checkpoint signaling.
• SOT evidence for PDCD1 SNPs remains weak and inconsistent.
• HSCT data associate PDCD1 variants with GVHD, relapse and survival.
• Donor–recipient checkpoint genetics may refine transplant risk.
• PDCD1 is a context-dependent modifier, not a validated standalone predictor.

## 1 Introduction

*The programmed cell death 1 gene (PDCD1)* encodes programmed death-1 protein (PD-1), an inhibitory immune checkpoint receptor that is induced after antigen exposure and is expressed on activated T cells, B cells, natural killer (NK) cells, dendritic cells, monocytes, and regulatory T cells [[1], [2], [3], [4], [5]]. Its main function is to place a physiological brake on immune activation, particularly when persistent antigenic stimulation could otherwise drive excessive inflammation or tissue injury [[6], [7], [8], [9]]. After binding to programmed death-ligand 1 (PD-L1) or programmed death-ligand 2 (PD-L2), PD-1 weakens T-cell receptor (TCR) and co-stimulatory signaling, reducing effector-cell expansion, cytokine production, and immune-mediated damage [[6], [7], [8], [9]]. This regulatory role is not a secondary feature of immunity; rather, it is central to peripheral tolerance, as disruption of PD-1 signaling has been linked to autoimmune manifestations and uncontrolled immune pathology in experimental models [[6], [7], [8], [9]]. In transplantation, where the immune system must distinguish between harmful foreignness and tolerable donor tissue, the PD-1 pathway occupies a particularly important position [[10], [11], [12], [13], [14], [15]].

Transplant-related alloimmunity has different clinical expressions according to transplant setting. In solid organ transplantation (SOT), donor-antigen recognition by recipient immune cells is usually discussed in terms of graft rejection, including acute cellular rejection, antibody-mediated rejection, mixed rejection, delayed graft dysfunction, and chronic allograft injury [[16], [17], [18], [19]]. In contrast, allogeneic hematopoietic stem cell transplantation (allo-hematopoietic stem cell transplantation (HSCT)), bone marrow transplantation (BMT), and cord blood transplantation (CBT) are not rejection models in the same clinical sense. In these settings, donor-derived immune cells may attack recipient tissues and produce graft-versus-host disease (GVHD), while the same donor immune response may also mediate beneficial graft-versus-leukemia effects; therefore, clinical outcome studies often report GVHD, relapse, cytomegalovirus infection, mortality, survival, and engraftment rather than rejection as defined in SOT [[20], [21], [22], [23], [24], [25], [26]]. Throughout our article, rejection is therefore used mainly for SOT endpoints, whereas alloimmune transplant outcomes are used as the broader term that also includes GVHD and other HSCT/CBT endpoints.

The connection between *PDCD1* and alloimmune transplant outcomes becomes more intriguing when inherited genetic variation is considered [[27], [28], [29], [30]]. Single-nucleotide polymorphisms (SNPs) in *PDCD1* may influence promoter activity, transcriptional regulation, messenger RNA (mRNA) stability, receptor expression, or linkage with broader immune-regulatory haplotypes [[27], [28], [29], [30]]. Even small differences in PD-1 pathway activity could shift the threshold at which alloreactive T cells are activated, restrained, exhausted, or maintained in a damaging inflammatory state [30,31]. For this reason, human studies have examined *PDCD1* polymorphisms in relation to acute rejection, delayed graft function, viral complications, immune quiescence, and stable graft outcomes after kidney and liver transplantation [[32], [33], [34], [35], [36], [37], [38]].

The central question of this review is therefore not whether *PDCD1* variation uniformly increases or decreases transplantation risk, but under which transplant-specific biological conditions a given variant becomes clinically consequential. In SOT, PD-1 activity is evaluated mainly in the context of recipient immune responses directed against donor tissue, whereas after allo-HSCT the donor-derived immune system can simultaneously mediate harmful GVHD and beneficial graft-versus-leukemia activity [10,20,39,40]. This difference in the direction and cellular origin of the alloimmune response means that the same *PDCD1* variant could plausibly have distinct, or even opposing, clinical consequences depending on whether it is carried by the donor or recipient, the immune compartment in which it acts, the inflammatory and immunosuppressive environment, and the endpoint being measured [23,26,36,41]. Accordingly, *PDCD1* variants are considered here as context-dependent modifiers of checkpoint activity rather than as uniformly harmful or protective alleles. Interpretation of the available evidence requires a clear distinction between genetic association, variant-specific function, causality, and clinical prediction. Associations between *PDCD1* genotypes and transplant outcomes identify potentially relevant loci but do not establish that the genotyped variant directly mediates the observed phenotype. Functional evidence, including altered transcription-factor binding or allele-dependent promoter activity, strengthens the biological plausibility of selected variants but remains insufficient to demonstrate a causal contribution to transplant outcomes without corresponding evidence in transplant-relevant immune compartments. Similarly, associations identified within individual cohorts, even when biologically plausible, cannot be considered clinically predictive until their discriminatory performance and reproducibility are demonstrated in independent or prospective populations.

Using this context-dependent framework, this review first outlines the biological rationale and structural basis for *PDCD1* variation, then evaluates direct human evidence separately in SOT and allo-HSCT/BMT/CBT, and finally examines indirect and translational evidence, sources of heterogeneity, and priorities for future study.

## 2 Literature search and study selection

A structured literature search was performed to identify studies addressing *PDCD1* polymorphisms, PD-1/PD-L1 signaling, and transplant-related immune outcomes. PubMed/MEDLINE, Scopus, Web of Science Core Collection, and Google Scholar were searched from database inception to 5 May 2026. The search strategy combined gene- and pathway-related terms with transplantation-related terms, including: “PDCD1”, “PD-1”, “programmed cell death 1”, “PD-L1”, “CD274”, “polymorphism”, “single-nucleotide polymorphism”, “SNP”, “rs36084323”, “rs5839828”, “rs11568821”, “rs2227981”, “rs2227982”, “rs10204525”, “transplant rejection”, “solid organ transplantation”, “kidney transplantation”, “liver transplantation”, “hematopoietic stem cell transplantation”, “bone marrow transplantation”, “cord blood transplantation”, “graft-versus-host disease”, “GVHD”, “relapse”, “engraftment”, and “cytomegalovirus infection”. Reference lists of relevant articles were also screened manually to identify additional eligible studies. Peer-reviewed human studies evaluating donor- or recipient-derived PDCD1 genotypes in relation to defined transplant outcomes were considered the principal source of direct clinical evidence. Functional studies of PDCD1 variants, pathway-level expression studies, and checkpoint-blockade reports were included when they provided mechanistic or translational context. Non-transplant studies were considered only when they provided variant-specific functional evidence relevant to PDCD1 biology and were treated as indirect rather than direct transplant evidence. Vascularized composite allotransplantation, including hand transplantation, was not included as a separate evidence category because the present review focused on transplant settings with available *PDCD1* donor- or recipient-genotype outcome studies. Therefore, any PD-1/PD-L1 literature outside kidney transplantation, liver transplantation, allo-HSCT, BMT, or CBT was considered only as general pathway-level context when directly relevant, rather than as hand-transplant-specific evidence. Functional studies of *PDCD1* variants, pathway-level expression studies, and checkpoint-blockade reports were included only when they provided mechanistic or translational context for interpreting the PD-1/PD-L1 axis in transplantation. Non-transplant disease studies were not treated as direct transplant evidence and were used only as indirect, hypothesis-generating evidence when they clarified variant function or PD-1 pathway biology. Articles without direct relevance to *PDCD1*/PD-1/PD-L1 biology, non-peer-reviewed materials, and studies without accessible methodological or outcome information were excluded. Eligible studies were subsequently classified according to transplant setting, donor or recipient genotype source, PDCD1 variant, clinical endpoint, and type of evidence before narrative synthesis. Because the available studies differed substantially in transplant setting, ancestry, variant selection, donor-recipient structure, and clinical endpoints, the evidence was synthesized narratively rather than by meta-analysis. The variant-focused evidence base comprised 14 core reports, including 12 primary human studies evaluating donor- or recipient-derived PDCD1 genotypes in relation to transplant outcomes, one meta-analysis, and one functional promoter study. Among the primary studies, two reports from an Iranian kidney-transplant cohort examined overlapping PDCD1 variants in relation to distinct clinical outcomes [34,42]. Both analyses were derived from the same cohort of 172 recipients and thus represent complementary assessments of the same study population rather than independent replication. Additional pathway-level, non-transplant functional, biomarker, and checkpoint-blockade reports were included only as mechanistic or translational context. Because a prospectively maintained, deduplicated Preferred Reporting Items for Systematic Reviews and Meta-Analyses (PRISMA)-style screening log was unavailable, the exact numbers of records identified, screened, and excluded cannot be reliably reconstructed and are therefore not reported.

## 3 Biological rationale: PD-1/PD-L1 in alloimmunity

PD-1 protein, encoded by the *PDCD1* gene, is a central inhibitory receptor that appears after lymphocyte activation and helps prevent immune responses from progressing beyond a controlled and tissue-preserving level [1,4,5]. Its ligands, PD-L1 and PD-L2, are expressed by antigen-presenting cells and can also be induced on several non-hematopoietic tissues during inflammation, allowing this pathway to regulate immune activity both systemically and within target organs [7,10,[43], [44], [45], [46]]. This regulatory role is particularly relevant in transplantation, where donor-recipient antigenic differences create a persistent stimulus for alloreactive T cells [47]. If such stimulation is not adequately restrained, activated T cells may expand, produce inflammatory mediators, enter transplanted tissues, and contribute to rejection or GVHD [39,47].

The biological effect of PD-1 is not simply immune suppression in a broad sense, but rather the careful adjustment of T-cell activity according to the strength and duration of antigenic stimulation [10]. After binding to PD-L1 or PD-L2, PD-1 reduces signals generated through the TCR and CD28 costimulatory pathway, thereby limiting pathways involved in proliferation, cytokine release, survival, and metabolic activation [48,49]. This checkpoint therefore acts at a point where alloreactive T cells are deciding whether to expand into a damaging effector population or remain under regulatory control [48]. In the transplant setting, even small changes in this threshold may be biologically meaningful because alloantigen exposure is often continuous and occurs in a highly inflammatory environment [10].

PD-L1 expression inside the graft provides an additional and more local form of immune regulation [10,50]. Endothelial cells, epithelial cells, stromal cells, and infiltrating immune cells can express PD-L1, especially under the influence of inflammatory cytokines such as interferon-γ [10,47,51]. Through this mechanism, the graft is not merely a passive target of immune injury, but can actively deliver inhibitory signals to infiltrating T cells [[50], [51], [52]]. Experimental transplantation studies have shown that interruption of PD-1/PD-L1 signaling can accelerate allograft damage, increase cellular infiltration, and weaken tolerance-promoting mechanisms [11,50,52]. Moreover, findings from cardiac and intestinal allograft models support the idea that tissue-associated PD-L1 helps contain early inflammatory amplification and limits the progression of rejection-related injury [15,51].

A similar principle applies in allo-HSCT, although the clinical balance is more complex [39,53,54]. In this context, donor T cells are needed for graft-versus-leukemia activity, but the same immune response can also damage host tissues and drive GVHD [39,53]. PD-1/PD-L1 signaling helps restrain excessive donor T-cell alloreactivity and therefore contributes to the control of immune-mediated tissue injury after transplantation [39,53,54]. At the same time, excessive checkpoint activity may reduce antitumor immunity, which explains why therapeutic PD-1 blockade after allo-HSCT can be associated with both clinical benefit and serious immune complications [53,54]. Thus, the pathway lies at a biologically delicate intersection between protective immunity and harmful alloimmune aggression [39,53,54].

These observations provide the biological basis for investigating *PDCD1* variation in transplantation. The phenotypic consequences of individual variants are likely to differ according to their genomic location and regulatory context. Regulatory effects have been demonstrated for selected *PDCD1* loci, whereas other reported associations may reflect linked variants or haplotypes rather than an independent effect of the genotyped SNP [37,42,55]. The gene architecture, functional annotations, and supporting experimental evidence for the principal *PDCD1* variants are considered next (Fig. 1).

**Fig. 1:**
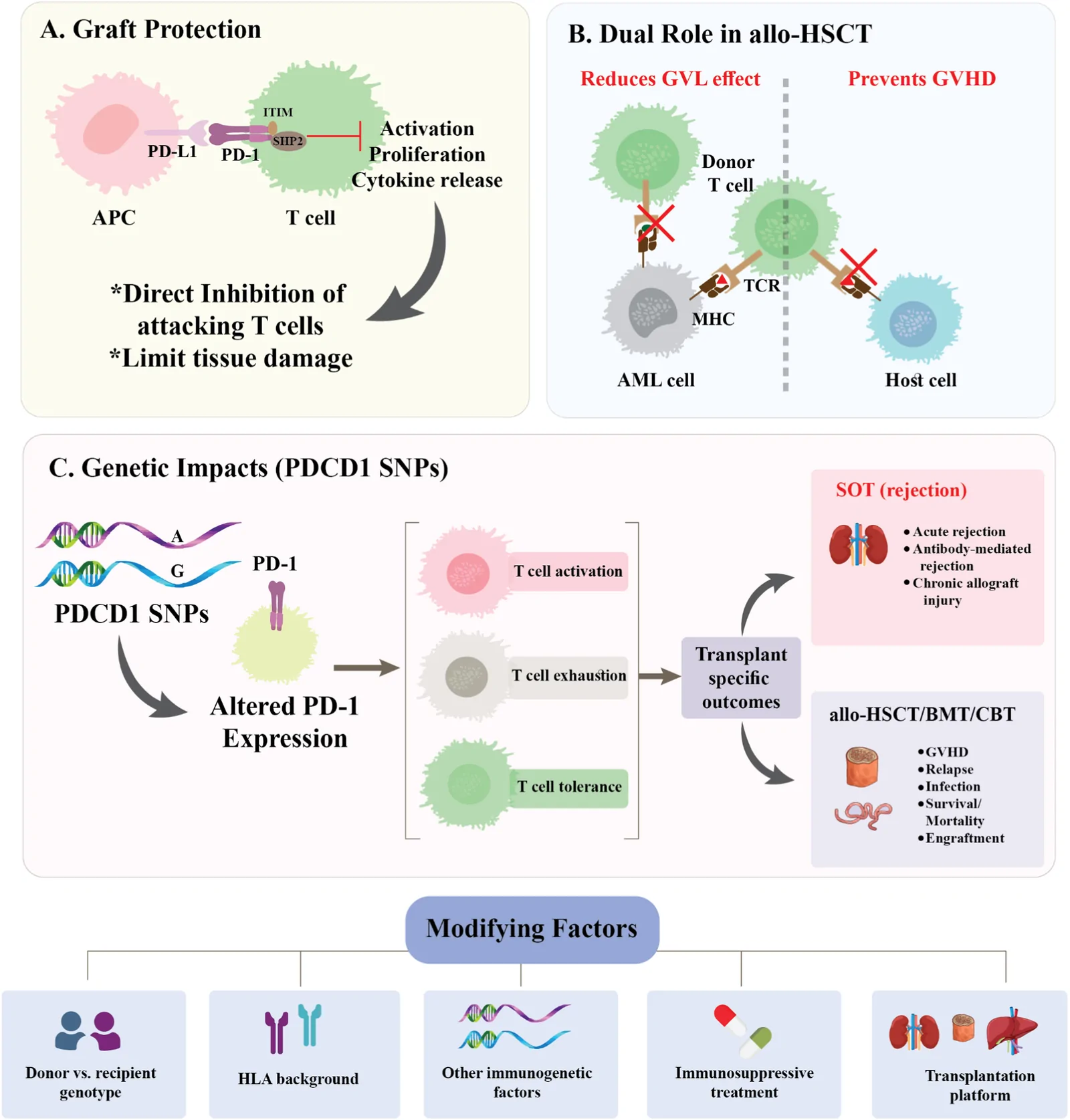
Multifaceted role of the PD-1/PD-L1 axis in T cell regulation and transplantation. (A) PD-1/PD-L1 signaling attenuates T cell activation by inhibiting TCR and CD28-mediated pathways, leading to reduced proliferation, cytokine production, and survival. (B) In allogeneic hematopoietic stem cell transplantation, this pathway exerts a dual role by limiting graft-versus-host disease (GVHD) while potentially compromising the beneficial graft-versus-leukemia (GVL) effect. (C) PDCD1 variation may influence checkpoint regulation through promoter activity, transcription-factor binding, RNA-level mechanisms, coding effects, or linkage with neighboring regulatory alleles. The strength of experimental support differs among individual variants, ranging from directly demonstrated regulatory activity to primarily association-based functional hypotheses.

## 4 *PDCD1* gene and protein architecture, SNP classes, and functional hypotheses

The current National Center for Biotechnology Information (NCBI) Gene annotation (RS_2025_08, Genome Reference Consortium Human Build 38, patch 14 [GRCh38.p14]) reports a gene-level exon count of six for the *PDCD1* locus. This gene-level count should not be used as a transcript-specific exon or intron numbering system because *PDCD1* has multiple annotated transcripts. Variant descriptions in this review are therefore anchored to the Matched Annotation from NCBI and EMBL-EBI (MANE) Select NCBI Reference Sequence (RefSeq) transcript/protein pair NM_005018.3/NP_005009.2, corresponding to Ensembl transcript ENST00000334409.10. Within this transcript framework, rs11568821 lies in the intronic interval between transcript exons 4 and 5, historically termed intron 4. Current database records identify rs11568821 as a multiallelic site at NM_005018.3:c.627 + 189, including NM_005018.3:c.627 + 189G > A, corresponding to NC_000002.12:g.241851760C > T, and NM_005018.3:c.627 + 189G > C, corresponding to NC_000002.12:g.241851760C > G. Because historical PD-1.3/+7146 notation has not always distinguished these alternate alleles consistently, it is retained only as a legacy cross-reference in this review [[56], [57], [58]]. Earlier genomic organization studies likewise described the principal *PDCD1* transcript using a five-exon architecture [2,59].

The encoded PD-1 protein, also known as CD279 and indexed in the UniProt Knowledgebase (UniProtKB)/Swiss-Prot as Q15116, is a 288-amino-acid single-pass type I membrane glycoprotein of the immunoglobulin superfamily. Structurally, PD-1 contains an extracellular immunoglobulin variable (IgV)-like domain, a transmembrane region, and a cytoplasmic tail carrying tyrosine-based inhibitory signaling motifs. In particular, the cytoplasmic tail contains an immunoreceptor tyrosine-based inhibitory motif centered on Y223 and an immunoreceptor tyrosine-based switch motif centered on Y248, which support recruitment of inhibitory signaling complexes and suppression of T-cell activation, including CD28/TCR-linked signaling [48,59,60]. These gene- and protein-level features are important for interpreting *PDCD1* SNPs, because promoter, intronic, exonic, and 3′UTR variants may affect different biological layers, including transcription, mRNA stability, receptor expression, ligand-dependent signaling, and downstream checkpoint function.

Among the selected *PDCD1* variants discussed in this review, reference SNP identifiers (rsIDs) are used as the primary identifiers, and historical PD-1.x aliases are provided in parentheses only to help cross-map older immunogenetic literature with current variant nomenclature. rs36084323 (PD-1.1) lies in the *PDCD1* promoter region and may influence inducible *PDCD1* transcription during immune activation [23,30,41]. rs5839828 is a length-polymorphic poly-G insertion/deletion (indel) in the *PDCD1* promoter region, historically reported using informal descriptions such as C > del, G/del, or G6/G7. In the current Ensembl GRCh38 representation, the variant spans chr2:241,859,601–241,859,607 and has the forward-strand allele string GGGGGG/GGGGGGG/GGGGGGGG. Because this homopolymeric site contains multiple repeat-length alleles, the rsID and database-anchored allele string are more reliable identifiers than study-specific “G” or “deletion” labels [[56], [57], [58]]. Promoter-reporter experiments have demonstrated allele-dependent transcriptional activity, although the available assay data do not by themselves establish how each database-defined repeat-length allele affects endogenous *PDCD1* expression in primary immune cells [30].

Throughout this review, the term “*PDCD1* variants” is used as an inclusive designation for both single-nucleotide polymorphisms and the promoter poly-G indel rs5839828.

rs11568821, historically reported as PD-1.3 or +7146, lies within the intronic interval between exons 4 and 5 of the *PDCD1* MANE Select transcript, historically designated intron 4, and has been proposed to alter runt-related transcription factor 1 (RUNX1)-dependent transcriptional regulation of *PDCD1* in activated lymphocytes [28]. The functional interpretation of *PDCD1* associations is further complicated by linkage disequilibrium across the locus. In the original characterization of PD-1.3, associations involving the variants historically designated PD-1.5 and PD-1.6 were largely explained by their linkage with PD-1.3 [28]. Thus, association signals within PDCD1 may arise from the genotyped variant itself, from a linked regulatory allele, or from a broader haplotypic background. This distinction is particularly relevant when functional data are unavailable for the specific SNP evaluated in a transplant cohort.

Other intronic *PDCD1* polymorphisms have also been investigated as potential regulators of PDCD1 transcription in autoimmune disease [61]. Because rs11568821 is multiallelic, historical allele labels should not be assumed to identify a single modern Human Genome Variation Society (HGVS) alternate allele without study-specific verification [[56], [57], [58]]. rs41386349, historically reported as *PDCD1* +7209C > T, is also relevant for nomenclature reconciliation because it was genotyped together with rs11568821 in the liver-transplant cohort of Thude et al.; however, it has not been repeatedly evaluated across transplant cohorts and is therefore discussed mainly in the context of that study [32].

Database-based annotation using the database of single-nucleotide polymorphisms (dbSNP) Build 157 and Ensembl Variant Effect Predictor release 116 on GRCh38.p14, anchored to the *PDCD1* MANE Select RefSeq transcript/protein pair NM_005018.3/NP_005009.2, classifies rs2227981 as a synonymous variant. For the commonly studied forward-strand A/G comparison, NC_000002.12:g.241851121A > G corresponds to NM_005018.3:c.804T > C and NP_005009.2:p.Ala268 = [[56], [57], [58]]. However, rs2227981 is a multiallelic site, with the Ensembl forward-strand allele string A/C/G/T. The historical +7785C > T (PD-1.5) convention reverses the reference/alternate assignment relative to the current MANE record: the allele historically labelled T corresponds to the MANE transcript reference allele NM_005018.3:c.804T and the GRCh38 forward-strand genomic A allele, whereas the allele historically labelled C corresponds to transcript c.804C and genomic G. Accordingly, allele-specific findings reported under legacy PD-1.5 nomenclature should be interpreted only after confirming the strand orientation and allele coding used in the original study.

Within the same annotation framework, rs2227982 is defined as NC_000002.12:g.241851281G > A, corresponding to NM_005018.3:c.644C > T and NP_005009.2:p.Ala215Val, and is classified as a missense variant [[56], [57], [58]]. Thus, rs2227981 does not alter the encoded alanine residue at position 268, whereas rs2227982 substitutes alanine with valine at residue 215. These database-defined consequence classes should be distinguished from experimentally demonstrated functional effects: possible effects of rs2227981 on RNA processing, translation, expression, or linked regulatory haplotypes remain hypothesis-generating, and the missense classification of rs2227982 alone does not establish altered PD-1 signaling [[62], [63], [64]]. Finally, rs10204525, sometimes reported as PD-1.6 in the literature, is located in the 3′ untranslated region (3′UTR) and may influence allele-specific microRNA-mediated regulation of *PDCD1* expression [65].

The potential biological effects of *PDCD1* variation are likely to depend on the regulatory consequence of the underlying allele. Variants that reduce or delay PD-1 induction could favor greater clonal expansion, cytokine production, and tissue infiltration by alloreactive T cells, whereas increased or sustained checkpoint activity could contribute to T-cell exhaustion, infection susceptibility, or attenuation of graft-versus-leukemia activity after allo-HSCT [8,23,40]. The strength of this mechanistic interpretation differs among individual variants. In liver transplantation, recipient *PDCD1* rs11568821 and donor CD274 rs4143815 were jointly associated with late acute rejection, supporting the relevance of coordinated variation across the PD-1/PD-L1 axis [36]. However, functional expression data in that study were generated for donor CD274 rs4143815 rather than recipient *PDCD1* rs11568821. Donor *PDCD1* rs36084323 and rs11568821 have also been associated with acute GVHD after human leukocyte antigen (HLA)-identical sibling allo-HSCT [41], while additional allo-HSCT studies have reported associations involving rs36084323, rs2227982, and other immune-regulatory variants [23]. These observations support a context-dependent genetic contribution to transplant outcomes, although the underlying signal may reflect direct variant function, linkage disequilibrium, or broader haplotypic effects. The principal variants and their functional evidence are summarized in Table 1.

**Table 1:** Nucleotide-level description and functional classes of selected *PDCD1* variants relevant to transplant immunology.

Variant (alias)	RS/nucleotide description	Genomic/functional location	Variant-specific functional evidence	Potential mechanistic relevance to transplantation	Ref.
rs36084323 (PD-1.1)	Database-anchored genomic HGVS on GRCh38: NC_000002.12:g.241859444C > T. Earlier literature commonly described this promoter variant as −606G > A or NM_005018.2.-606G > A. Because the latter description is based on a superseded transcript version and is not a current MANE Select NM_005018.3 expression, it is retained only as a legacy transcript-oriented cross-reference.	*PDCD1* promoter/upstream regulatory region.	Allele-dependent transcriptional activity has been demonstrated in promoter-reporter assays, supporting a potential regulatory effect on *PDCD1* transcription	The demonstrated promoter activity provides a plausible regulatory basis for reported transplant associations; however, the clinical signal may also be influenced by haplotypic context or linked regulatory variation.	Database annotation [[56], [57], [58]]; functional and association context [30,63,66]
rs5839828	Database-anchored Ensembl representation on GRCh38: chr2:241,859,601–241,859,607; forward-strand allele string GGGGGG/GGGGGGG/GGGGGGGG. This is a multiallelic, length-polymorphic poly-G indel. Informal descriptions such as C > del, G/del, or G6/G7 are retained only as study-specific legacy cross-references and should not be interpreted as universal reference/alternate allele assignments.	*PDCD1* promoter region; historically reported as the −763 promoter variant.	Variation in the length of the promoter poly-G tract may alter *PDCD1* promoter activity. In the original dual-luminescence reporter study, the construct labelled as the del allele showed 1.37 ± 0.24 times the transcriptional activity of the construct labelled as the G allele; however, these informal assay labels should not be directly equated with every repeat-length allele recorded in current databases.	Allele-dependent promoter activity supports a regulatory role for this locus, although its contribution to HSCT outcomes remains context dependent and may also reflect linked regulatory or haplotypic effects.	Database annotation [[56], [57], [58]]; HSCT association evidence [23]; functional promoter-reporter evidence [30].
rs11568821 (PD-1.3)	Historical literature notation: PD-1.3, +7146G > A, or 7146G-A. Current database records identify rs11568821 as a multiallelic site at NM_005018.3:c.627 + 189, including NM_005018.3:c.627 + 189G > A [NC_000002.12:g.241851760C > T] and NM_005018.3:c.627 + 189G > C [NC_000002.12:g.241851760C > G]. Historical notation is retained only as a legacy cross-reference and should not be assumed to specify one modern alternate allele without verification of the original study.	Intronic enhancer-like regulatory region between exons 4 and 5 of the MANE Select transcript NM_005018.3 (historically termed intron 4).	Experimental evidence supports altered RUNX1 binding within this intronic regulatory region, with potential effects on *PDCD1* transcriptional regulation in activated lymphocytes	The experimentally supported regulatory effect provides biological plausibility for transplant associations involving this locus. However, the contribution of rs11568821 to rejection or GVHD may depend on transplant context, donor–recipient origin, and linkage with neighboring *PDCD1* variants.	[28,33,41,59]
rs2227981 (PD-1.5)	Legacy literature notation: +7785C > T (PD-1.5). Current database records identify rs2227981 as a multiallelic site with the GRCh38 forward-strand allele string A/C/G/T. For the commonly studied A/G comparison, the database-anchored HGVS expressions are NC_000002.12:g.241851121A > G, NM_005018.3:c.804T > C, and NP_005009.2:p.Ala268 = . The legacy C > T convention reverses the reference/alternate assignment relative to the MANE Select transcript: the legacy T allele corresponds to NM_005018.3:c.804T and genomic A, whereas the legacy C allele corresponds to transcript c.804C and genomic G. See the table footnote for interpretation.	Coding region of the MANE Select transcript; synonymous variant (Sequence Ontology: SO:0001819).	Synonymous substitution at codon 268 with no change in the encoded alanine residue (p.Ala268 = ). Any biological effect would therefore require a mechanism other than amino-acid substitution, such as linkage disequilibrium with regulatory variation, altered RNA processing, translational efficiency, or context-dependent PD-1 expression.	Transplant relevance remains indirect and hypothesis-generating because the synonymous consequence itself does not establish altered PD-1 expression or signaling; any association may also reflect RNA-level regulation or linkage with another functional allele.	Database annotation [[56], [57], [58]]; functional or association context [[62], [63], [64],^67^]
rs2227982 (PD-1.9)	Legacy literature notation: +7625C > T (PD-1.9). Database-anchored HGVS on GRCh38/MANE Select: NC_000002.12:g.241851281G > A; NM_005018.3:c.644C > T; NP_005009.2:p.Ala215Val.	Coding region of the MANE Select transcript; missense variant (Sequence Ontology: SO:0001583).	Missense substitution replacing alanine with valine at residue 215 of the MANE Select PD-1 protein (NP_005009.2:p.Ala215Val). The database-defined missense consequence establishes a protein-sequence change but does not, by itself, demonstrate an effect on PD-1 expression, structure, ligand binding, or inhibitory signaling.	The p.Ala215Val substitution provides a plausible basis for functional investigation, but its effect on PD-1 signaling has not been established. Reported transplant associations should therefore be interpreted independently of the missense annotation and may also reflect linked variation.	Database annotation [[56], [57], [58]]; transplant or functional context [63,64].
rs10204525 (sometimes reported as PD-1.6)	Database-anchored HGVS on GRCh38/MANE Select: NC_000002.12:g.241850169C > T and NM_005018.3:c.*889G > A. Because *PDCD1* is located on the reverse genomic strand, the genomic C > T substitution corresponds to the transcript-level G > A substitution. The historical +8669G > A notation is retained only as a legacy cross-reference.	3′ untranslated region of the *PDCD1* MANE Select transcript NM_005018.3.	May affect post-transcriptional regulation, including allele-specific microRNA interaction and *PDCD1* mRNA stability.	Altered 3′UTR regulation could influence sustained PD-1 expression during chronic antigen exposure, with possible relevance to immune exhaustion, viral immune control, and late alloimmune regulation; direct transplant-specific evidence remains limited.	Database annotation [[56], [57], [58]]; functional context [65,68,69]

**Footnote:** Variant consequences and HGVS expressions were verified on 26 July 2026 against NCBI dbSNP Build 157 and Ensembl Variant Effect Predictor release 116 using the GRCh38.p14 assembly and the *PDCD1* MANE Select RefSeq transcript/protein pair NM_005018.3/NP_005009.2 [[56], [57], [58]]. Consequence terms follow Sequence Ontology. Because *PDCD1* is located on the reverse genomic strand, the GRCh38 forward-strand genomic alleles differ from the corresponding transcript-level alleles: rs2227981 is NC_000002.12:g.241851121A > G and NM_005018.3:c.804T > C (NP_005009.2:p.Ala268 = ), whereas rs2227982 is NC_000002.12:g.241851281G > A and NM_005018.3:c.644C > T (NP_005009.2:p.Ala215Val). Historical PD-1.x nucleotide descriptions are retained only as legacy cross-references and should not be used to infer the current reference/alternate allele or protein consequence. Exon and intron numbering is transcript specific. The designation “intron 4” for rs11568821 refers to the intronic interval between exons 4 and 5 of the *PDCD1* MANE Select transcript NM_005018.3/ENST00000334409.10 and should not be inferred directly from the NCBI gene-level exon count. rs11568821 is a multiallelic rsID. The historical PD-1.3/+7146 notation has been used inconsistently in older reports and is therefore retained only as a legacy cross-reference; current HGVS alleles are stated explicitly when they can be verified. For rs36084323, NC_000002.12:g.241859444C > T is used as the current GRCh38 database anchor, whereas NM_005018.2.-606G > A and −606G > A are retained only as legacy transcript-oriented descriptions. For rs10204525, NC_000002.12:g.241850169C > T corresponds to NM_005018.3:c.*889G > A because *PDCD1* is located on the reverse genomic strand.

Abbreviations: GVHD, graft-versus-host disease; HGVS, Human Genome Variation Society; PD-1, programmed death-1 protein; PDCD1, programmed cell death 1 gene; RUNX1, runt-related transcription factor 1; SNP, single-nucleotide polymorphism; 3′UTR, 3′ untranslated region.

**Note:** For rs2227982, p.Ala215Val/A215V is used as the standardized reference-to-variant protein description; the reverse term Val215Ala appears in some older clinical studies and should be interpreted cautiously.

**Note:** For rs2227981, +7785C > T (PD-1.5) is a legacy allele convention rather than a current reference-sequence HGVS description. The site is multiallelic. The allele designated T in the legacy literature corresponds to NM_005018.3:c.804T, the MANE Select transcript reference allele, and NC_000002.12:g.241851121A on the GRCh38 forward strand. Conversely, the legacy C allele corresponds to transcript c.804C and genomic g.241851121G. Therefore, study-reported allele effects should be interpreted according to the original assay coding and strand orientation and should not be extrapolated to the other alternate alleles recorded under this rsID.

**Note:** rs5839828 is a multiallelic length polymorphism within a homopolymeric poly-G tract rather than a conventional single-nucleotide substitution. Ensembl represents the GRCh38 forward-strand alleles as GGGGGG/GGGGGGG/GGGGGGGG at chr2:241,859,601–241,859,607. Earlier studies used informal labels such as C > del, G/del, G6, and G7. These labels depend on the sequence orientation, repeat-length convention, and assay coding used in the original report and should not be treated as standardized HGVS reference/alternate descriptions. Allele-specific findings should therefore be interpreted using the original study methodology and reconciled with the database-defined repeat length whenever possible.

## 5 Direct human transplant-genetic evidence in solid organ transplantation

The *PDCD1* gene, which encodes the inhibitory immune-checkpoint receptor PD-1, has been investigated as a biologically relevant candidate locus in SOT because PD-1 protein regulates T-cell activation, peripheral tolerance, and alloimmune responses [32,33]. In this context, human studies have mainly examined whether *PDCD1* SNPs are associated with acute rejection, delayed graft function, viral complications, or stable graft outcomes after kidney and liver transplantation [32,33]. The principal human studies evaluating *PDCD1*-related polymorphisms in SOT are summarized in Table 2.

**Table 2:** Direct human transplant-genetic evidence for *PDCD1* SNPs in solid organ transplantation.

Transplant setting	Study design/population	*PDCD1* variant(s) evaluated	Main *PDCD1*-related findings	Evidence type/strength	Clinical endpoint(s)	Ref.
Liver transplantation	Retrospective study in liver transplant recipients with and without acute rejection	*PDCD1* rs11568821 (PD-1.3; historically reported as +7146G > A) and rs41386349 (+7209C > T); CTLA4 rs733618 and rs4553808	No significant association between the tested *PDCD1* or CTLA4 variants and acute liver allograft rejection in German recipients.	Direct recipient genotype-outcome evidence; negative association; low strength because it is based on a single retrospective cohort and has not been independently replicated.	Acute rejection	[32]
Liver transplantation	Cohort of 528 liver-transplant recipients and 410 donors; five *PDCD1*/CD274 SNPs were genotyped and associations with early and late acute rejection were evaluated.	Recipient *PDCD1* rs11568821 and donor CD274/PD-L1 rs4143815	Recipient rs11568821 A-allele carrier status was associated with late acute rejection in multivariable analysis (HR 3.739; p = 0.004). The highest risk was observed in A-allele recipients receiving grafts from rs4143815 GG donors.	Direct recipient-donor genotype-outcome evidence with pathway-level functional support; moderate strength but based on a single cohort without independent replication.	Late acute rejection	[36]
Kidney transplantation	Retrospective study in kidney allograft recipients classified as biopsy-proven acute rejection or stable graft function	rs11568821 (PD-1.3; study-reported as +7146G > A) and rs2227982 (PD-1.9; study-reported as +7625C > T)	The genotype and allele frequencies of *PDCD1* rs11568821 and rs2227982 did not differ significantly between the acute-rejection and stable-graft-function groups. However, rs2227982 (PD-1.9) was more frequent among recipients with delayed graft function, including those with both delayed graft function and acute rejection.	Direct recipient genotype-outcome evidence; endpoint-dependent signal; low-to-moderate strength because the finding was more apparent for delayed graft function than for acute rejection alone and requires replication.	Acute rejection; delayed graft function	[33]
Kidney transplantation	Cohort study of 172 kidney-transplant recipients, including 45 recipients with acute rejection and 127 without acute rejection	rs11568821 (PD-1.3; study-reported as +7146G > A) and rs2227982 (PD-1.9; study-reported as +7625C > T)	No significant overall differences in *PDCD1* genotype or allele frequencies were observed between recipients with and without acute rejection. Subgroup analyses identified sex- and donor-type-dependent associations, including lower frequencies of rs2227982 CT/TT genotypes among male recipients with acute rejection and of the rs11568821 GA genotype among male acute-rejection recipients of deceased-donor grafts. No *PDCD1*-specific association with patient survival was identified.	Direct recipient genotype-outcome evidence; low strength because the *PDCD1* associations were subgroup-specific, originated from a single cohort, and have not been independently replicated.	Acute rejection; survival	[42]
Kidney transplantation	Cohort study of 469 kidney-graft recipients evaluating genetic susceptibility to post-transplant cytomegalovirus infection.	rs11568821 (PD-1.3)	The rs11568821 A allele was associated with an increased risk of cytomegalovirus infection among seropositive recipients who did not receive Cytomegalovirus prophylaxis (OR 2.60; p = 0.006) and remained independently associated with Cytomegalovirus infection in multivariable analysis (OR 2.54; p = 0.010).	Direct recipient genotype-clinical outcome evidence; low-to-moderate strength because the association was derived from a single cohort and requires independent replication.	Cytomegalovirus infection	[55]
Kidney transplantation	A cohort of kidney transplant recipients was evaluated for co-stimulatory gene polymorphisms and viral outcomes	rs11568821 (PD-1.3; study-reported A/G alleles)	A higher frequency of the rs11568821 (PD-1.3) genotype and allele was reported among cytomegalovirus-infected female recipients. The study did not identify rs11568821 as the principal rejection-related signal.	Direct genotype-clinical outcome evidence for cytomegalovirus infection; indirect/weak evidence for rejection because the *PDCD1*-related signal was mainly subgroup- and infection-associated.	Viral infection-related outcome; acute rejection context	[34]
Kidney transplantation	Non-concurrent cohort of 100 Egyptian kidney-transplant recipients, including 50 recipients with biopsy-confirmed acute rejection and 50 recipients without acute rejection.	*PDCD1* rs2227982	The rs2227982 AG genotype was similarly distributed between acute-rejection and no-rejection groups, with no significant *PDCD1*-specific association with acute rejection or graft survival.	Direct recipient genotype-outcome evidence; negative association; low-to-moderate strength because it is based on a single cohort.	Acute rejection; graft survival	[70]
Solid organ transplantation (meta-analysis; mainly kidney and liver studies)	Meta-analysis of co-stimulatory molecule gene polymorphisms in allograft rejection	*PDCD1* polymorphisms included, not individually detailed in the study	The meta-analysis evaluated *PDCD1* among candidate immune-regulatory genes, but the study highlighted stronger associations for CD28 and CD86 rather than a distinct *PDCD1*-specific rejection signal.	Secondary synthesis of candidate-gene evidence; low *PDCD1*-specific strength because *PDCD1* was not independently validated as the dominant predictor.	Acute allograft rejection	[35]

**Abbreviations:***PDCD1*, programmed cell death 1 gene; PD-1, programmed death-1 protein/receptor; G, guanine; A, adenine; C, cytosine; T, thymine.

NoteReferences [34] and [39] were derived from the same cohort of 172 Iranian kidney-transplant recipients and should therefore be interpreted as complementary analyses of an overlapping study population rather than as independent replication.

In liver transplantation, the available direct evidence is limited and largely negative. Thude et al. evaluated two cytotoxic T-lymphocyte-associated protein 4 (CTLA4) variants, rs733618 and rs4553808, and two *PDCD1* variants, rs11568821 (PD-1.3; historically +7146G > A) and rs41386349 (historically +7209C > T), in German liver transplant recipients with and without acute rejection [32]. They found no significant differences in genotype or allele frequencies between recipients with acute rejection and those without acute rejection. Notably, rs41386349 was evaluated in this liver-transplant cohort [32], but it has not been independently replicated as a *PDCD1*-associated transplant marker.

Zolfaghari et al. examined polymorphisms in *PDCD1* and *CD274* (encoding PD-L1; reported as *PDCD1LG1* in the original study) in kidney allograft recipients with biopsy-proven acute rejection or stable graft function [33]. Overall, the studied *PDCD1* and *CD274* genotype and allele frequencies did not differ significantly between the acute-rejection and stable-graft-function groups. However, the *PDCD1* rs2227982 (PD-1.9) genotype was more frequent among transplant recipients than among healthy controls and was enriched in patients with delayed graft function, including the subgroup with both delayed graft function and acute rejection [33]. These findings suggest that *PDCD1* variation may be more relevant to early graft injury or delayed graft function than to acute rejection alone.

Viral immune regulation has also been examined in relation to *PDCD1* polymorphisms after kidney transplantation. Hoffmann et al. evaluated *rs11568821* (*PD-1.3*) in 469 kidney-graft recipients and found that the A allele was associated with an increased risk of cytomegalovirus infection among seropositive recipients who did not receive *Cytomegalovirus* prophylaxis; the association remained significant after multivariable adjustment [55]. In a separate report from the 172-recipient Iranian cohort described above, Niknam et al. found a higher frequency of the *rs11568821* genotype and allele among cytomegalovirus-infected female recipients [34]. These findings support a possible role for *PDCD1* variation in post-transplant viral immune control, although both associations remain population- or subgroup-dependent and require independent replication. A broader meta-analysis of co-stimulatory molecule gene polymorphisms and acute allograft rejection found stronger rejection-related associations for *CD28* and *CD86* than for *PDCD1* [35].

The SOT evidence summarized in Fig. 2 includes a negative liver-transplant association study [32], a recipient-donor PD-1/PD-L1 interaction associated with late acute rejection in a separate liver-transplant cohort [36], endpoint- or subgroup-specific kidney-transplant findings involving acute rejection, delayed graft function, or cytomegalovirus infection [33,34,42,55], and a more recent negative association for rs2227982 [70]. None of these PDCD1 findings has yet been independently replicated in a directly comparable cohort.

**Fig. 2:**
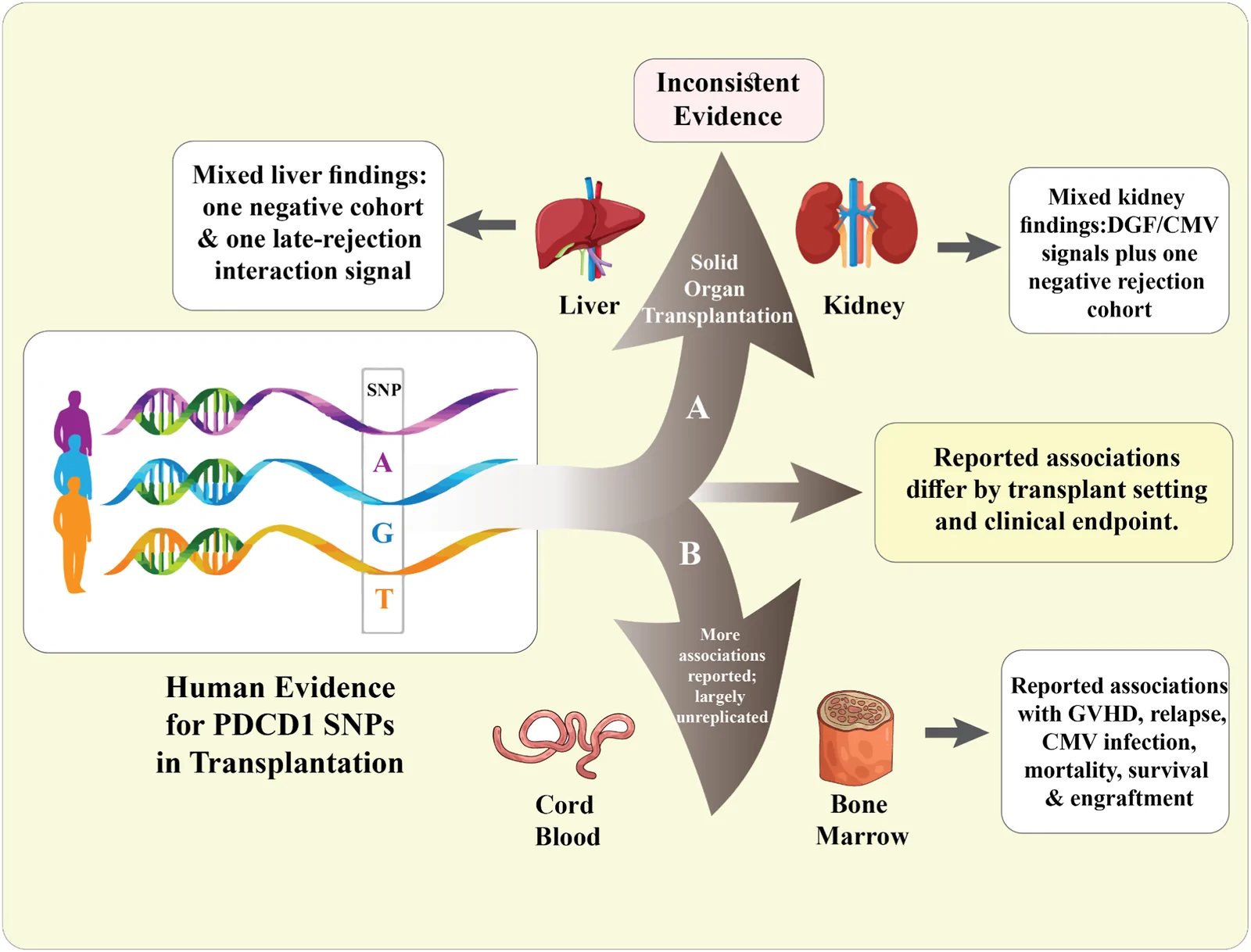
Summary of PDCD1 SNP associations in transplantation. (A) In solid organ transplantation, evidence for PDCD1 SNPs as predictors of acute rejection is weak, with only possible links to delayed graft function and viral complications. (B) In HSCT/CBT, PDCD1 variants are more consistently associated with adverse outcomes including GVHD, relapse, infection, and mortality. PDCD1 is best understood within a broader multi-gene immune framework.

## 6 Direct human transplant-genetic evidence after allo-HSC, bone marrow, and cord blood transplantation

*PDCD1* gene polymorphisms have also been investigated after allo-HSCT, BMT, and CBT [[23], [24], [25], [26]]. Because the graft in these settings is hematopoietic and immune-cell derived, the clinically relevant endpoints differ from those used in SOT rejection, where the injured target is a transplanted solid organ [[20], [21], [22]]. Therefore, this section does not treat GVHD, relapse, cytomegalovirus infection, mortality, survival, or engraftment as forms of SOT-like rejection; rather, these endpoints are discussed as HSCT/CBT alloimmune or transplant-effectiveness outcomes that may be influenced by non-HLA immune-regulatory variation [[23], [24], [25], [26]]. The main human studies evaluating *PDCD1*-related polymorphisms in these transplantation settings are summarized in Table 3.

**Table 3:** Direct human transplant-genetic evidence for *PDCD1* variants after allogeneic hematopoietic stem cell, bone marrow, and cord blood transplantation.

Transplant setting	Study design/population	*PDCD1* variant(s) evaluated	Main *PDCD1*-related findings	Evidence type/strength	Clinical endpoint(s)	Ref.
HSCT	Study of patients with acute leukemia undergoing HSCT and their respective donors	Five *PDCD1* variants reported as significantly associated with at least one post-HSCT outcome: rs5839828, rs36084323 (PD-1.1), rs41386349, rs6705653, and rs2227982 (PD-1.9)	The five *PDCD1* variants were associated with one or more adverse post-HSCT outcomes, including mortality, cytomegalovirus infection, GVHD, and relapse. The direction and clinical endpoint of the associations differed according to the variant, leukemia subtype, and genetic model.	Direct donor/recipient genotype-outcome evidence; exploratory multi-gene association; low-to-moderate strength pending independent validation.	Mortality; cytomegalovirus infection; graft-versus-host disease; relapse	[23]
Allogeneic HSCT from HLA-identical sibling donors	Multicenter donor-genotype study including 1485 HLA-identical sibling-donor allo-HSCT procedures.	Donor rs36084323 (PD-1.1) and rs11568821 (PD-1.3)	Donor rs36084323 GG was associated with an increased risk of grade II-IV acute GVHD (HR 2.2; p = 0.033), and donor rs11568821 AA was associated with a higher risk of grade II-IV acute GVHD (HR 4.5; p < 0.001). No association was reported with overall survival or relapse.	Direct donor genotype-outcome evidence from a large multicenter cohort; moderate strength, although independent replication remains required.	Grade II-IV acute GVHD; overall survival; relapse	[41]
CBT	Study of donors in unrelated cord blood transplantation	rs36084323 (PD-1.1) and rs2227982 (PD-1.9)	Donor *PDCD1* variants were associated with selected CBT outcomes. A polygenic model incorporating non-HLA variants showed an AUC of 0.7692 within the study cohort, without independent validation.	Direct genotype–outcome association; exploratory within-cohort predictive modeling; no independent predictive validation.	Mortality; relapse; cytomegalovirus infection; graft-versus-host disease; engraftment effectiveness	[26]
Allogeneic HSCT from HLA-identical sibling donors	Multicenter donor-genotype study	*PDCD1* rs36084323 (PD-1.1), analyzed in combination with LAG3 rs870849	When analyzed together with donor LAG3 genotype, *PDCD1* rs36084323 helped define genetic groups associated with differential transplant outcomes.	Direct donor genotype-outcome evidence in a combined LAG3-*PDCD1* model; moderate biological relevance but limited *PDCD1*-specific independence.	Acute graft-versus-host disease; transplant-related mortality; overall survival; disease-free survival	[24]
Haploidentical HSCT	Study of patients and donors undergoing haploidentical HSCT	rs5839828 (promoter poly-G indel) and rs36084323 (PD-1.1)	*PDCD1* rs5839828 and rs36084323 were significantly related to adverse outcomes after haploidentical HSCT.	Direct genotype-outcome evidence; preliminary/low strength because of the exploratory cohort design and need for replication.	Graft-versus-host disease; survival status; relapse	[25]
HSCT-related functional evidence	Functional promoter-reporter study of *PDCD1* promoter variants previously linked to HSCT and/or autoimmune disease	rs5839828 (promoter poly-G indel) and rs36084323 (PD-1.1)	Both *PDCD1* promoter variants showed allele-dependent transcriptional activity in reporter assays, providing functional support for their possible regulatory effects. For rs5839828, the allele labels used in the original assay were study-specific and should be reconciled with the current database-defined poly-G repeat lengths.	Functional evidence only; mechanistic support for promoter activity but not direct clinical genotype-outcome evidence.	Functional effect on gene expression	[30]

**Abbreviations:** HSCT, hematopoietic stem cell transplantation; BMT, bone marrow transplantation; CBT, cord blood transplantation; HLA, human leukocyte antigen; *PDCD1*, programmed cell death 1 gene; PD-1, programmed death-1 protein/receptor; SNP, single-nucleotide polymorphism; LAG3, lymphocyte activation gene 3.

**Note:** Variants not included in Table 1 are presented in Table 3 using the rs identifiers and nomenclature reported in the original studies.

In patients with acute leukemia undergoing HSCT, Chen et al. examined non-HLA co-stimulatory gene variants in both recipients and donors and reported that several *PDCD1* SNPs were associated with adverse post-transplant outcomes, including mortality, cytomegalovirus infection, GVHD, and relapse [23]. These findings placed *PDCD1* within a broader panel of immune-regulatory genes, including *CTLA4*, *CD28*, and *tumor necrosis factor superfamily member 4 (TNFSF4)*, that may contribute to post-HSCT alloimmune and infectious complications [23].

Evidence from unrelated CBT further supports the potential relevance of non-HLA immune-regulatory polymorphisms. Chen et al. investigated donor SNPs in immune-regulatory genes and found that two *PDCD1* variants were associated with transplantation effectiveness and adverse post-transplant reactions, including mortality, relapse, cytomegalovirus infection, GVHD, and engraftment-related outcomes [26]. The study additionally developed a polygenic risk model based on selected non-HLA variants, with an area under the receiver operating characteristic curve of 0.7692 in the study cohort. Because model performance was assessed within the same limited cohort and independent validation was unavailable, these findings provide preliminary evidence of discriminatory potential rather than established clinical predictive utility [26].

Other HSCT studies have evaluated *PDCD1* as part of combined donor checkpoint-genotype profiles. Cruz et al. focused primarily on donor *lymphocyte activation gene 3 (LAG3)* genotype in HLA-identical sibling transplantation but included *PDCD1* rs36084323 in a combined genetic model [24]. When donor *LAG3* rs870849 and *PDCD1* rs36084323 were analyzed together, distinct genotype groups were associated with acute GVHD, transplant-related mortality, overall survival, and disease-free survival [24]. Similarly, Tseng et al. reported that *PDCD1* rs5839828 and rs36084323, together with variants in other transplantation-related genes, were significantly associated with adverse outcomes after haploidentical HSCT, including GVHD, survival status, and relapse [25].

An important feature of the current allo-HSCT evidence is the predominance of donor-genotype signals. Donor *PDCD1* rs36084323 and rs11568821 were associated with acute GVHD in a large HLA-identical sibling cohort, while additional studies identified donor *PDCD1* associations with relapse, cytomegalovirus infection, GVHD, mortality, or engraftment-related outcomes across conventional HSCT, CBT, and haploidentical transplantation [[23], [24], [25], [26],^41^]. This donor-weighted pattern is biologically consistent with the transfer of donor-derived lymphocytes and hematopoietic progenitors, which progressively establish the post-transplant immune compartment and mediate both graft-versus-host and graft-versus-leukemia responses [[20], [21], [22]]. Recipient genotype remains mechanistically relevant, particularly during the early inflammatory phase in which residual host antigen-presenting cells and recipient tissues participate in donor T-cell activation and tissue injury. The relative contribution of donor and recipient *PDCD1* variation may therefore differ according to immune-cell origin, timing after transplantation, graft source, and the clinical endpoint under study.

Functional studies provide an additional layer for interpreting *PDCD1* genotype–outcome associations. Chen et al. demonstrated allele-dependent transcriptional activity for the promoter-region variants rs5839828 and rs36084323 using reporter assays [30]. Although these findings provide direct evidence of allele-dependent regulatory activity, they do not establish that the observed transcriptional effects mediate the clinical associations reported for GVHD, relapse, or other post-transplant outcomes. These findings support a regulatory role for both loci and provide a plausible molecular basis for their reported associations in HSCT cohorts. The magnitude and direction of these effects in primary transplant-relevant immune cells, however, remain incompletely characterized, and linked regulatory variation may also contribute to the observed clinical signals. Accordingly, rs5839828 and rs36084323 currently have stronger variant-specific functional support than *PDCD1* loci for which evidence is based primarily on genotype-outcome associations.

## 7 Indirect and translational evidence: pathway-level data, rs2227981, and checkpoint blockade

After direct transplant-genetic studies, indirect evidence should be considered separately because it does not carry the same clinical weight as donor-recipient SNP association studies. Three forms of indirect evidence are particularly relevant for *PDCD1*: pathway-level expression or biomarker studies in transplantation, variant-specific data from non-transplant immune-mediated diseases or cancer immunotherapy, and clinical perturbation of the PD-1/PD-L1 axis by checkpoint blockade. These data strengthen biological plausibility, but they should not be interpreted as direct proof that a given *PDCD1* SNP predicts rejection, GVHD, relapse, or graft survival.

### 7.1 Pathway-level expression and biomarker studies versus SNP evidence

Several kidney-transplant studies have assessed *PDCD1* at the pathway or expression level rather than at the inherited SNP level. For example, bioinformatic analysis identified *PDCD1* as a potential biomarker related to acute rejection after kidney transplantation, and immune-quiescence gene-expression studies have evaluated transplant immune status using transcriptomic signatures rather than germline *PDCD1* genotypes [37,38]. These studies are useful because they show that the PD-1 axis may be active in transplant immune states, but they should be discussed separately from SNP association studies. Gene expression, immune-cell exhaustion, biomarker performance, and inherited genetic variation represent different biological layers and should not be treated as interchangeable evidence.

### 7.2 Variant-specific indirect evidence: rs2227981 (PD-1.5)

The *PDCD1* rs2227981 polymorphism, also known as PD-1.5, should be interpreted as an indirect and hypothesis-generating candidate rather than as an established transplant-rejection marker. Unlike promoter or enhancer SNPs that may directly alter transcriptional activity, rs2227981 is a synonymous variant located in exon 5 of the MANE Select transcript NM_005018.3, and its potential biological relevance may instead involve linkage disequilibrium with regulatory variants, altered mRNA processing, translational efficiency, or context-dependent effects on PD-1 expression. Available transplant-focused evidence does not yet demonstrate that rs2227981 independently predicts acute rejection, GVHD, graft loss, or survival.

In advanced melanoma patients treated with PD-1 inhibitors, rs2227981 was evaluated together with other PD-1/PD-L1 variants, but the tested variants were not clearly associated with therapeutic response. In that study, the allele reported as the PD-1.5 T allele under the legacy +7785C > T convention, corresponding to NM_005018.3:c.804T, the MANE Select reference allele, and NC_000002.12:g.241851121A on the GRCh38 forward strand, showed only a trend toward a lower risk of immune-related adverse events [71]. This observation is conceptually relevant because both immune-related adverse events and transplant rejection involve disturbance of checkpoint-regulated tolerance. However, immune toxicity after cancer immunotherapy cannot be directly equated with transplant rejection. Therefore, rs2227981 should remain in the manuscript as a variant of interest, but its evidence level should be clearly labelled as indirect until transplant-specific donor-recipient cohorts validate its role.

### 7.3 Checkpoint blockade as a clinical perturbation of the PD-1/PD-L1 axis

Checkpoint blockade provides an important translational context because it clinically perturbs the same PD-1/PD-L1 axis that is genetically examined in *PDCD1* SNP studies. In cancer, PD-1 inhibition can restore antitumor immunity, whereas in transplantation, reduced inhibitory checkpoint signaling may intensify alloimmune activation and tissue injury. This duality is especially visible after allo-HSCT, where PD-1 blockade for relapsed lymphoma can produce antitumor responses but has also been associated with frequent or severe GVHD and immune toxicity [53,54]. Checkpoint blockade therefore serves as a pathway-level perturbation model for interpreting PD-1 biology in transplantation.

## 8 Sources of heterogeneity and evidence interpretation framework

The strength of the available evidence differs substantially across PDCD1 variants and should be interpreted accordingly. Genotype–outcome studies provide evidence of association, whereas experimental studies demonstrating altered transcription-factor binding or promoter activity provide variant-specific functional support [28,30]. These evidence levels are complementary but not equivalent: functional activity does not establish that a variant causally determines a transplant phenotype, and statistical association alone provides no evidence of clinical predictive performance. At present, the literature supports transplant-related associations for several PDCD1 loci and regulatory effects for selected variants, but direct causal mediation and independently validated predictive utility remain unproven [23,26,28,30,41].

A useful conceptual model is to consider PDCD1 variation within the immune compartment, transplantation setting, and clinical endpoint in which a genotype–outcome association is observed, while also accounting for the strength of variant-specific functional evidence. In SOT, the dominant alloimmune effector response is typically recipient-derived and directed against donor tissue; PD-1 signaling on recipient T cells and PD-L1 expression within the graft can restrain this response, providing a biological framework in which reduced checkpoint activity could favor graft-directed effector responses and rejection [10,36,50]. Human liver-transplant data illustrate this compartment-specific interaction, as recipient PDCD1 rs11568821 and donor CD274 rs4143815 were jointly associated with susceptibility to late acute rejection [36]. This genetic interaction is consistent with involvement of the PD-1/PD-L1 axis, although the association itself does not establish that rs11568821 directly alters PD-1 expression or signaling. By contrast, after allo-HSCT, donor-derived immune cells repopulate the recipient, making donor PDCD1 genotype particularly relevant to the checkpoint-regulatory background of transferred immune cells [41]. In this setting, variation associated with stronger PD-1-mediated restraint could theoretically limit GVHD while attenuating graft-versus-leukemia activity, whereas variation associated with weaker checkpoint restraint could favor antitumor immunity at the cost of greater GVHD; experimental and clinical perturbation of the PD-1/PD-L1 axis supports the biological plausibility of this trade-off [39,40,53,54]. However, for transplant-associated SNPs without direct functional characterization, the underlying signal may arise from the tested allele itself, a linked functional variant, or a broader haplotypic background. Thus, GVHD and relapse can represent opposing clinical manifestations of variation within the same checkpoint-regulatory pathway rather than endpoints expected to show a uniform direction of genetic association. Infection and engraftment require more cautious interpretation because they are additionally shaped by immune reconstitution, conditioning, graft source, pathogen exposure, and immunosuppressive treatment. Associations between PDCD1 variants and these endpoints should therefore be regarded as more context dependent and mechanistically less direct than associations with predominantly T-cell-driven alloimmune phenotypes [23,26]. Consequently, the direction and magnitude of a PDCD1 association should be interpreted in relation to the available variant-specific functional evidence, donor-versus-recipient genotype, transplant setting, immune environment, clinical endpoint, and potential linkage with other regulatory variants rather than as an intrinsic property of the associated allele itself. This framework provides a biological explanation for why the same PDCD1 marker may appear favorable for one transplantation outcome, unfavorable for another, or clinically neutral in a different setting.

The heterogeneity of *PDCD1* findings is best understood by separating the type of evidence being evaluated. Direct transplant-genetic evidence includes studies linking donor or recipient *PDCD1* variants to defined transplant outcomes. In SOT, this evidence remains limited and inconsistent: a German liver-transplant cohort did not find a significant association between tested *PDCD1* polymorphisms and acute rejection, whereas a kidney-transplant study suggested that *PDCD1* variation may be more relevant to delayed graft function than to acute rejection alone [32,33]. In contrast, allo-HSCT and CBT studies more often report associations between *PDCD1* or broader non-HLA immune-regulatory SNPs and outcomes such as GVHD, relapse, cytomegalovirus infection, engraftment, mortality, and survival [23,26].

A second source of heterogeneity is the uneven level of functional characterization across *PDCD1* variants. The promoter-region variants rs5839828 and rs36084323 have shown allele-dependent transcriptional activity in reporter assays [30], whereas rs11568821 has been linked to altered RUNX1 binding within an intronic regulatory region [28]. These variants therefore have direct experimental support for regulatory activity. In contrast, the biological interpretation of rs2227981 and rs2227982 relies more heavily on sequence annotation, disease-association studies, and indirect functional observations. For such loci, the observed genetic signal may reflect subtle effects on RNA processing, translation, protein function, or linkage with another regulatory allele. Differences in linkage-disequilibrium structure across ancestry groups may further modify which marker captures the underlying functional haplotype.

A third source of heterogeneity is the donor-recipient structure of transplantation, which is particularly important in allo-HSCT because the cellular origin of the immune compartment changes after transplantation. The graft introduces donor-derived mature lymphocytes and hematopoietic progenitors, and subsequent immune reconstitution progressively places the PDCD1 genotype of the donor within T-cell and other hematopoietic compartments that participate in post-transplant immunity. These donor-derived cells mediate both GVHD and graft-versus-leukemia activity, providing a biological basis for the association of donor PDCD1 genotypes with alloimmune outcomes [[20], [21], [22],^41^]. By contrast, recipient-derived antigen-presenting cells and inflammatory tissue compartments can contribute to the initiation of donor T-cell responses, particularly during the early post-transplant period [[20], [21], [22]]. Thus, donor and recipient PDCD1 genotypes represent biologically distinct exposures rather than interchangeable measures of the same genetic effect. The relevance of each may vary with the stage of immune reconstitution, graft source, conditioning intensity, immunosuppression, and the endpoint being evaluated.

Available human studies illustrate this compartment-specific structure but remain incomplete. Donor rs36084323 and rs11568821 genotypes have been associated with acute GVHD after HLA-identical sibling allo-HSCT [41], and donor PDCD1 variants have also been associated with relapse, infection, engraftment, and other outcomes in HSCT and CBT cohorts [[23], [24], [25], [26]]. In SOT, an analogous cross-compartment interaction has been observed between recipient PDCD1 rs11568821 and donor CD274 rs4143815 in late acute liver-graft rejection [36], demonstrating that checkpoint-related genetic effects can depend on the cellular and tissue origin of the interacting components. For allo-HSCT, the net phenotype may similarly reflect donor genotype, recipient genotype, and their interaction rather than either genotype in isolation. Paired studies that genotype both members of the transplant pair and prespecify donor-only, recipient-only, and donor×recipient interaction models would therefore provide a more informative framework for resolving these effects. Integration with longitudinal chimerism, immune-cell-specific PD-1 expression, and variant-specific functional assays could further determine whether a genetic association tracks with the donor-derived effector compartment, the recipient environment, or their interaction.

A fourth source of heterogeneity is the broader immunogenetic background in which PDCD1 variation is evaluated. The clinical effect associated with a PDCD1 marker is unlikely to be independent of other determinants of alloimmune activation. In allo-HSCT, the degree and locus-specific pattern of HLA compatibility strongly influence donor–recipient allorecognition and the risks of GVHD, graft failure, relapse, and mortality, providing a major genetic context within which non-HLA associations should be interpreted [[20], [21], [22],^72^]. NK -cell regulation introduces an additional layer through donor killer-cell immunoglobulin-like receptor (KIR) genotype and donor KIR–recipient HLA ligand combinations, which can modify NK-cell alloreactivity and have been associated with post-transplant relapse and survival, although the direction and magnitude of these effects vary among transplantation platforms and predictive models [73]. Other checkpoint and co-stimulatory loci may also contribute to the same clinical phenotypes. PDCD1 has been evaluated together with *CTLA4*, *CD28*, *TNFSF4*, *inducible T-cell costimulator (ICOS)*, and *LAG3* in transplant cohorts, and combined genotype analyses have identified associations that were not attributable to PDCD1 alone [23,24,32,42]. For example, donor PDCD1 rs36084323 contributed to outcome stratification when analyzed jointly with donor LAG3 rs870849 [24], illustrating how a PDCD1 signal may depend on the surrounding immune-regulatory genotype. *CTLA4* variation has likewise been associated with GVHD, relapse, and survival after allo-HSCT in independent cohorts [74]. These observations indicate that PDCD1 associations may be modified by, or statistically confounded with, HLA compatibility, KIR–HLA interactions, and variation in other immune-checkpoint pathways. Consequently, future PDCD1 studies should incorporate these variables as prespecified covariates or interaction terms rather than interpreting individual PDCD1 loci in isolation.

A fifth source of heterogeneity is the immunosuppressive regimen used before and after transplantation. The phenotypic expression of a PDCD1-associated genetic effect is unlikely to be independent of pharmacologic immune suppression because these treatments modify many of the same T-cell activation, proliferation, differentiation, and immune-reconstitution processes regulated by the PD-1 pathway [75]. In allo-HSCT, calcineurin inhibitor-based prophylaxis is commonly combined with methotrexate or other agents, whereas alternative platforms may incorporate sirolimus, mycophenolate mofetil, or post-transplant cyclophosphamide; corticosteroids remain central to the treatment of established GVHD [75]. These regimens are not immunologically equivalent. For example, sirolimus/tacrolimus and methotrexate/tacrolimus have produced different rates of acute GVHD and different patterns of regulatory T-cell reconstitution [76], while a randomized phase III trial demonstrated superior GVHD-free, relapse-free survival with post-transplant cyclophosphamide, tacrolimus, and mycophenolate mofetil compared with tacrolimus/methotrexate prophylaxis [77]. Immunosuppressive heterogeneity is also relevant in SOT, where calcineurin inhibitors, corticosteroids, antiproliferative agents, and mechanistic target of rapamycin (mTOR) inhibitors differ in their mechanisms and intensity of immune suppression [78]. Consequently, differences in drug class, combination, exposure, timing, and treatment of post-transplant complications may modify or obscure associations between PDCD1 genotype and GVHD, rejection, infection, relapse, or survival. Future genetic studies should therefore report immunosuppressive protocols in sufficient detail and incorporate regimen, treatment exposure, and, where sample size permits, genotype-by-treatment interactions into the analysis.

Population-specific allele-frequency differences provide an additional explanation for ancestry-dependent heterogeneity. Allele frequencies were obtained from the 1000 Genomes Project Phase 3 final integrated variant release (release 20130502, version 5), as provided through Ensembl release 116, and were accessed on 26 July 2026 [79]. Frequencies are reported for the East Asian (EAS) and European (EUR) super-populations. Alleles are stated in the 5′-to-3′ orientation of the *PDCD1* MANE Select transcript NM_005018.3; because *PDCD1* is located on the reverse genomic strand, these transcript-oriented alleles are complementary to the corresponding GRCh38 forward-strand genomic alleles. The approximate alternative-allele frequencies in EAS versus EUR were 0.47 versus 0.02 for the rs36084323 A allele, 0.45 versus 0.02 for the rs2227982 T allele, and approximately 0.00 versus 0.12 for the rs11568821 c.627 + 189G > A alternate allele, which corresponds to NC_000002.12:g.241851760C > T on the GRCh38 forward strand [79]. rs10204525 also showed population-dependent allele-frequency differences. These differences do not establish functional causality but may influence statistical power, linkage-disequilibrium structure, donor-recipient genotype distributions, and the probability of detecting a candidate association in a given ancestry group. Cross-ancestry replication and ancestry-aware modeling are therefore required before a *PDCD1* association is interpreted as broadly generalizable [23,25,79].

Despite the biological plausibility of PDCD1 involvement in alloimmune regulation, translation of individual variants into clinically useful risk markers remains limited. Transplant outcomes are multifactorial, and even a statistically significant single-locus association may provide only a modest incremental contribution to individual risk discrimination when considered alongside HLA compatibility, KIR–HLA interactions, other immune-regulatory loci, and established clinical determinants [24,[72], [73], [74]]. Generalizability is further restricted by differences in ancestry and allele frequency, transplant platform, graft source, conditioning intensity, immunosuppressive exposure, and endpoint definition across cohorts [[23], [24], [25], [26],^41^,[75], [76], [77],^79^]. In allo-HSCT, donor and recipient PDCD1 genotypes also represent biologically distinct exposures, and their effects may depend on donor–recipient genotype combinations and interactions with other checkpoint or co-stimulatory genes rather than on an isolated PDCD1 locus [24,41,74]. Statistical multiplicity represents an additional concern because several candidate-gene studies evaluated multiple variants, inheritance models, and clinical outcomes relative to cohort size. This limitation is particularly relevant to small exploratory datasets, including the haploidentical HSCT study that assessed 75 SNPs in 24 donor–recipient pairs [25]. Approaches to multiple testing have also differed among studies: the CBT analysis applied Bonferroni correction, whereas the *LAG3/PDCD1* study explicitly acknowledged the absence of adjustment for multiple comparisons [24,26]. Finally, most reported PDCD1 associations have not been reproduced in independent, directly comparable transplant cohorts. Collectively, these limitations reduce the reproducibility, transportability, and predictive value of individual PDCD1 associations and currently preclude their use as standalone markers for donor selection or individualized risk stratification.

A comprehensive variant-level comparison of the available transplant studies, integrating genomic location, study population, donor or recipient genotype source, sample size, clinical endpoint, effect direction and magnitude, statistical significance, replication status, and available functional evidence, is provided in Table 4. Taken together, the inconsistent literature is more plausibly explained by context dependence than by a uniform effect of *PDCD1* variation. Associations should therefore be interpreted within prespecified transplant settings and endpoints, with explicit consideration of donor versus recipient genotype, ancestry, immunosuppression, infection, and interactions with HLA and other immune-regulatory loci. This framework also requires strict separation of germline SNP associations from expression-based or pharmacologic evidence and independent validation in adequately powered donor-recipient cohorts.

**Table 4:** Comprehensive variant-level summary of *PDCD1* polymorphisms evaluated across transplant settings.

*PDCD1* variant	Genomic location	Population/cohort	Donor or recipient genotype	Sample size	Transplantation type	Clinical endpoint	Direction and magnitude of association	Statistical significance	Replication status	Available functional evidence	Ref.
rs11568821 (PD-1.3)	Intronic regulatory region between transcript exons 4 and 5; historically intron 4	German/Caucasian cohort	Recipient; study-reported 7146G > A	204 recipients: 100 acute rejection, 104 without rejection	Liver transplantation	Acute rejection	No significant difference in genotype or allele frequencies between rejection and non-rejection groups	NS	No positive endpoint-matched replication; later LT study reported an association specifically with late acute rejection	Experimental non-transplant evidence supports altered RUNX1 binding at this regulatory locus [28]; transplant-specific mediation remains unconfirmed	[32]
rs41386349	Intronic region; historically +7209C > T	German/Caucasian cohort	Recipient	204 recipients	Liver transplantation	Acute rejection	No significant genotype or allele association	NS	No independent replication for acute liver rejection	No direct variant-specific transplant functional evidence	[32]
rs11568821 (PD-1.3)	Intronic regulatory region	Erasmus MC liver-transplant cohort, the Netherlands	Recipient AA/AG vs GG; evaluated together with donor CD274 rs4143815	528 recipients; 410 donors genotyped	Liver transplantation	Late acute rejection (>6 months)	Recipient A-allele carriage associated with increased risk; HR 3.739	p = 0.004	Single positive LT cohort; not directly replicated with the same endpoint definition	Functional analysis in this study concerned donor CD274 rs4143815, rather than functional alteration by recipient rs11568821; independent RUNX1-related evidence exists for rs11568821 [28]	[36]
rs11568821 (PD-1.3)	Intronic regulatory region	French kidney-transplant cohort	Recipient A allele	469 recipients	Kidney transplantation	CMV infection	A allele associated with increased CMV risk in seropositive recipients without prophylaxis: OR 2.60; adjusted OR 2.54	p = 0.006; adjusted p = 0.010	A CMV-related association was also observed in an Iranian cohort, but in a sex-specific subgroup and with different allele patterns; therefore, consistent allelic replication is lacking	Variant-specific regulatory evidence derives from non-transplant studies rather than this transplant cohort	[55]
rs11568821 (PD-1.3)	Intronic regulatory region	Iranian cohort	Recipient	122 recipients; 61 AAR and 61 SGF	Kidney transplantation	Acute rejection	No significant difference between AAR and SGF groups	p > 0.05	Consistent with other negative/heterogeneous kidney-rejection findings; no uniform rejection signal	No functional assay performed in this cohort	[33]
rs2227982 (PD-1.9)	Coding region; missense p.Ala215Val	Iranian cohort	Recipient; study-reported +7625C > T	122 recipients; 61 AAR and 61 SGF	Kidney transplantation	Acute rejection; delayed graft function	No significant AAR-versus-SGF association; genotype/T allele enriched among recipients with delayed graft function; 27/122 recipients developed DGF	AAR vs SGF: NS; DGF association reported as significant	DGF-specific finding has not been independently replicated; rejection findings remain inconsistent	Missense annotation establishes an amino-acid substitution, but altered PD-1 signaling in transplant-relevant cells has not been established	[33]
rs11568821 (PD-1.3)	Intronic regulatory region	Iranian cohort	Recipient	172 recipients; 45 acute rejection, 127 without rejection	Kidney transplantation	Acute rejection	No overall association; GA genotype showed a sex-dependent subgroup association with lower acute-rejection frequency in males	Overall NS; subgroup p = 0.03	Same population contributed to related viral-outcome analysis; subgroup observation lacks independent replication	No variant-specific functional assay in this cohort	[42]
rs2227982 (PD-1.9)	Coding region; p.Ala215Val	Iranian cohort	Recipient	172 recipients	Kidney transplantation	Acute rejection	No overall association; CT and TT genotypes showed sex-dependent subgroup differences	Overall NS; subgroup p = 0.03 and p = 0.04	Subgroup finding not independently replicated	Direct transplant-specific functional evidence unavailable	[42]
rs11568821 (PD-1.3)	Intronic regulatory region	Iranian cohort	Recipient	172 recipients; 31 with active CMV infection	Kidney transplantation	CMV infection	GG genotype and G allele were more frequent among CMV-infected female recipients	Genotype p = 0.02; allele p = 0.005	Direction differs from the A-allele CMV signal reported in the French cohort; therefore, no consistent allelic replication	No functional assay in this cohort	[34]
rs2227982 (PD-1.9)	Coding region; p.Ala215Val	Egyptian cohort	Recipient; AG genotype evaluated	100 recipients: 50 biopsy-confirmed AR, 50 without AR	Kidney transplantation	Acute rejection; graft survival	AG genotype equally distributed between rejection and non-rejection groups; no *PDCD1*-specific association	NS	Independent negative kidney-transplant cohort; does not support a consistent rejection association	No direct functional assay; missense status alone does not establish altered PD-1 function	[70]
rs36084323 (PD-1.1)	Promoter/upstream regulatory region	Spanish multicenter cohort	Donor GG vs other genotypes	1485 donor–recipient pairs	HLA-identical sibling allo-HSCT	Grade II–IV acute GVHD	Donor GG genotype associated with increased GVHD risk: HR 2.2, 95% CI 1.1–4.8	p = 0.033	Additional hematopoietic-transplant associations reported, but with different endpoints and populations; no strict endpoint-matched replication	Reporter assay demonstrates allele-dependent promoter activity; rs36084323 T construct showed 0.68 ± 0.07-fold activity relative to C, p < 0.001 [30]	[30,41]
rs11568821 (PD-1.3)	Intronic regulatory region	Spanish multicenter cohort	Donor AA vs other genotypes	1485 donor–recipient pairs	HLA-identical sibling allo-HSCT	Grade II–IV acute GVHD	Donor AA genotype associated with increased GVHD risk: HR 4.5, 95% CI 2.0–10.1	p < 0.001	Strong single-cohort association; independent endpoint-matched replication remains unavailable	Non-transplant experimental evidence supports altered RUNX1 binding [28]; causal mediation of GVHD has not been demonstrated	[41]
rs41386349	Intronic region	Taiwanese acute-leukemia cohort	Donor	163 donor–recipient pairs	Allogeneic HSCT	Mortality; CMV infection; mild GVHD	AML mortality: recessive OR 2.62 (1.07–6.42); ALL CMV: allele OR 2.86 (1.08–7.56); also associated with mild GVHD	Mortality p = 0.0362; CMV allele p = 0.0356	No comparable independent replication; association differs by disease subtype and endpoint	No direct functional assay; LD with neighboring *PDCD1* variation complicates variant-specific attribution	[23]
rs6705653	Intronic region	Taiwanese acute-leukemia cohort	Donor	163 donor–recipient pairs	Allogeneic HSCT	CMV infection; GVHD	AML CMV dominant model: OR 7.91 (1.54–40.71); ALL CMV dominant model: OR 3.95 (1.25–12.49); also associated with mild/chronic GVHD in ALL	p = 0.0138 and p = 0.0201, respectively	No independent transplant replication	No direct variant-specific functional assay; linkage structure with nearby intronic *PDCD1* markers may contribute to the signal	[23]
rs5839828	Promoter poly-G length polymorphism	Taiwanese acute-leukemia cohort	Donor; study-specific G6/G7 repeat coding	163 donor–recipient pairs	Allogeneic HSCT	Relapse in ALL	G7 allele associated with higher relapse risk: OR 3.80 (1.75–8.48); additive model OR 4.02 (1.64–9.83)	Allele p = 0.0008; additive p = 0.0024	Relapse association subsequently observed in a small haplo-HSCT cohort, providing supportive but exploratory replication	Reporter assay demonstrates allele-dependent promoter activity; study-labelled del construct showed 1.37 ± 0.24-fold activity relative to G, p < 0.001 [30]	[23,30]
rs36084323 (PD-1.1)	Promoter	Taiwanese acute-leukemia cohort	Donor	163 donor–recipient pairs	Allogeneic HSCT	CMV infection; relapse in ALL	C allele: CMV OR 2.39 (1.11–5.15); relapse OR 2.80 (1.29–6.17)	CMV p = 0.0265; relapse p = 0.0095	Relapse-related signals subsequently reported in CBT and haplo-HSCT; endpoints and populations differ	Direct promoter-reporter evidence supports allele-dependent transcriptional activity [30]	[23,30]
rs2227982 (PD-1.9)	Coding region; p.Ala215Val	Taiwanese acute-leukemia cohort	Donor	163 donor–recipient pairs	Allogeneic HSCT	CMV infection; relapse; mild/chronic GVHD in ALL	G allele: CMV OR 2.53 (1.13–5.67); relapse OR 3.90 (1.67–9.35); mild GVHD OR 3.40 (1.44–8.03)	CMV p = 0.0252; relapse p = 0.0018; mild GVHD p = 0.0055	Relapse association also observed in CBT, but across a different graft source/population	Missense consequence is established; direct alteration of PD-1 signaling in transplant-relevant cells remains unconfirmed	[23]
rs36084323 (PD-1.1)	Promoter	Taiwanese unrelated CBT cohort, predominantly pediatric	Donor CC vs CT + TT	65 donors	Cord blood transplantation	Relapse	Dominant model associated with higher relapse risk: OR 4.210 (1.331–13.320)	p = 0.012	Supports a recurring relapse-related association across hematopoietic transplantation, although not strict replication	Allele-dependent promoter activity demonstrated independently [30]	[26,30]
rs2227982 (PD-1.9)	Coding region; p.Ala215Val	Taiwanese unrelated CBT cohort, predominantly pediatric	Donor GG vs AG + AA	65 donors	Cord blood transplantation	Relapse	Dominant model associated with higher relapse risk: OR 5.194 (1.612–16.739)	p = 0.004	Provides cross-setting support for the relapse signal reported after HSCT; direct replication remains limited	No transplant-specific demonstration that p.Ala215Val alters PD-1 signaling	[26]
rs10204525	3′UTR	Taiwanese unrelated CBT cohort	Donor	65 donors	Cord blood transplantation	Mortality	Recessive model OR 0.231 (0.056–0.951)	p = 0.033, nominal; did not meet the study's stricter multiplicity threshold	No independent transplant replication	Non-transplant evidence supports allele-specific post-transcriptional regulation; transplant-specific functional validation is unavailable	[26]
rs36084323 (PD-1.1)	Promoter	Taiwanese AML/ALL cohort	Donor	24 donor–recipient pairs	Haploidentical HSCT	Relapse	Significant association under the additive model; effect estimate not reported in the main-text summary	p = 0.042	Exploratory support for the relapse association previously observed after HLA-matched HSCT; very small cohort	Allele-dependent promoter activity demonstrated independently [30]	[25]
rs5839828	Promoter poly-G length polymorphism	Taiwanese AML/ALL cohort	Donor	24 donor–recipient pairs	Haploidentical HSCT	Relapse	Significant association under additive, recessive, and homozygous models; effect estimates not reported in the main-text summary	p = 0.014, p = 0.037, and p = 0.015	Supportive replication of the earlier relapse signal, but exploratory because of the very small cohort	Direct promoter-reporter evidence available [30]	[25]
rs36084323 (PD-1.1)	Promoter	Spanish multicenter cohort	Donor, analyzed jointly with LAG3 rs870849	797 recipients; both genotypes available for 732 donors	HLA-identical sibling allo-HSCT	Severe acute GVHD; OS; DFS; TRM	Combined LAG3–*PDCD1* genotype groups stratified outcomes; *PDCD1*-specific independent effect was not established in the multivariable combined analysis	Combined-group severe GVHD p = 0.025; *PDCD1*-specific TRM term p = 0.087	Supports context dependence and gene–gene interaction rather than independent replication of a *PDCD1*-specific effect	Direct promoter-regulatory evidence for rs36084323 exists independently [30]	[24]

**Abbreviations:** AAR, acute allograft rejection; ALL, acute lymphoblastic leukemia; AML, acute myeloid leukemia; CBT, cord blood transplantation; CI, confidence interval; CMV, cytomegalovirus; DFS, disease-free survival; DGF, delayed graft function; GVHD, graft-versus-host disease; HLA, human leukocyte antigen; HR, hazard ratio; HSCT, hematopoietic stem cell transplantation; LD, linkage disequilibrium; LT, liver transplantation; NR, not reported; NS, not statistically significant; OR, odds ratio; OS, overall survival; *PDCD1*, programmed cell death 1 gene; PD-1, programmed death-1; SGF, stable graft function; TRM, transplant-related mortality; 3′UTR, 3′ untranslated region.

**Note:** Effect estimates and statistical significance are presented according to the genetic model used in each original study. Replication status refers to independent evaluation of the same *PDCD1* variant in a sufficiently comparable transplantation setting and clinical endpoint. Associations observed in different transplant platforms, populations, or endpoints are considered supportive cross-setting evidence rather than strict replication. Functional evidence is distinguished from clinical genotype–outcome association; reporter activity, transcription-factor binding, or sequence annotation alone does not demonstrate that a variant causally mediates the corresponding transplant outcome. The promoter poly-G variant rs5839828 is represented using study-specific allele nomenclature where necessary because historical G/del or G6/G7 designations are not directly interchangeable with all current database-defined repeat-length alleles. Predictive models derived and evaluated within the same cohort were considered exploratory and were not regarded as clinically validated predictors in the absence of independent or prospective validation.

## 9 Conclusions and research priorities

Progress in this field now depends less on testing additional isolated *PDCD1* SNPs than on improving study design. Priority should be given to multicenter cohorts in which both donors and recipients are genotyped and analyzed using prespecified donor-only, recipient-only, and donor×recipient interaction models within standardized, transplant-specific endpoints. Such studies should incorporate ancestry-aware and haplotype-based analyses and account for major immunogenetic determinants, including HLA compatibility, KIR–HLA relationships, and variation in other checkpoint or co-stimulatory loci. Immunosuppressive regimens, conditioning strategies, treatment exposure, and major post-transplant therapeutic interventions should also be recorded systematically and incorporated as covariates or interaction terms where appropriate. Where feasible, longitudinal chimerism and immune-cell-specific functional measurements should be integrated to identify the cellular compartment underlying each association. Accordingly, future clinical translation should prioritize externally validated multivariable models that integrate *PDCD1* variation with donor–recipient immunogenetic, transplant-related, and treatment-related variables rather than relying on isolated SNP-based risk estimates. Current evidence therefore supports context-dependent associations between *PDCD1* variation and selected transplant outcomes, with variant-specific regulatory activity demonstrated for only a subset of loci. However, neither a causal contribution to transplant phenotypes nor independently validated predictive utility has been established for any individual *PDCD1* variant, and clinical application of *PDCD1* genotyping requires prospective validation in adequately powered, transplant-specific cohorts.

## CRediT authorship contribution statement

**Hosein Rabet:** Writing – review & editing, Visualization. **Sayed Mostafa Hosseini:** Writing – review & editing, Supervision, Conceptualization.

## Declaration of generative AI and AI-assisted technologies in the writing process

During the preparation of this work, the authors used ChatGPT and Grammarly for language editing and grammar improvement. After using these tools, the authors reviewed and edited the content as needed and take full responsibility for the content of the publication.

## Funding

This research received no external funding.

## Declaration of competing interest

The authors declare that they have no known competing financial interests or personal relationships that could have appeared to influence the work reported in this paper.

## Data Availability

No data were generated or analyzed during the current study.
